# Supplementary material for: Adverse pregnancy outcomes and long-term risk of maternal renal disease: a systematic review and meta-analysis protocol
Source: BMJ Open. 2019 May 5;9(5):e027180. doi: 10.1136/bmjopen-2018-027180 (PMC6502020; doi:10.1136/bmjopen-2018-027180)
Supplement: Supplementary data [file bmjopen-2018-027180supp001.pdf]

SUPPLEMENTARY FILE – Search Strategy

Preeclampsia & gestational hypertension

H1	Antenatal
H2	Ante-natal
H3	Prenatal
H4	Pre-natal
H5	Pregnan*
H6	Gestation*
H7	H1 OR H2 OR H3 OR H4 OR H5 OR H6
H8	Pre Eclampsia
H9	Preeclampsia
H10	Pre-eclampsia
H11	Eclampsia
H12	Edema-Proteinuria-Hypertension
H13	Edema Proteinuria Hypertension
H14	Oedema-Proteinuria-Hypertension
H15	Oedema Proteinuria Hypertension
H16	EPH
H17	Toxemia*
H18	Toxaemia*
H19	Gestosis
H20	(H19 OR H7)
H21	Toxemia* OR Toxaemia* or EPH
H22	H21 AND H20
H23	Gestational hypertension
H24	Pregnancy-induced hypertension
H25	Pregnancy induced hypertension
H26	Hypertensi*
H27	Blood pressure
H28	H26 OR H27
H29	H28 AND H7
H30	H8 OR H9 OR H10 OR H11 OR H12 OR H13 OR H14 OR H15 OR H22 OR H23 OR H24 OR H25 OR H29

Gestational diabetes

D1	Pregnancy-Induced diabetes
D2	Pregnancy Induced diabetes
D3	Gestational Diabetes
D4	diabetes in pregnancy
D5	diabetes of pregnancy
D6	Diabetes mellitus in pregnancy
D7	Diabetes mellitus of pregnancy
D8	pregnancy-related diabetes
D9	pregnancy related diabetes
D10	D1 OR D2 OR D3 OR D4 OR D5 OR D6 OR D7 OR D8 OR D9 OR D10

Low birth weight

W1	Low Birth Weight
W2	Low-Birth-Weight
W3	Low Birth Weights
W4	Low-Birth-Weights
W5	Low Birthweight
W6	Low Birthweights
W7	Extremely Low Birth Weight
W8	Extremely-Low-Birth-Weight
W9	Extremely-Low-Birth-Weights
W10	Extremely Low Birthweight
W11	Very Low Birth Weight
W12	Very-Low-Birth-Weight
W13	Very-Low-Birth-Weights
W14	Very Low Birthweight
W15	Birth Weights
W16	Birth Weight
W17	Birthweight
W18	LBW
W19	VLBW
W20	ELBW
W21	Small for gestational age
W22	Growth restrict*
W23	IUGR
W24	W1 OR W2 OR W3 OR W4 OR W5 OR W6 OR W6 OR W7 OR W8 OR W9 OR W10 OR W11 OR W12 OR W13 OR W14 OR W15 OR W16 OR W17 OR W18 OR W19 OR W20 OR W21 OR W22 OR W23

Preterm birth or SGA

P1	Premature Birth
P2	Preterm Birth
P3	Premature Births
P4	Preterm Births
P5	Pre-term Birth
P6	Pre-term Births
P7	Gestational age
P8	Gestational ages
P9	Fetal Age
P10	Foetal Age
P11	Fetal Ages
P12	Foetal Ages
P13	Premature Labor
P14	Preterm Labor
P15	Pre-term Labor
P16	Premature Labour
P17	Preterm Labour
P18	Pre-term Labour
P19	Premature Obstetric Labor
P20	Premature Obstetric Labour
P21	P1 OR P2 OR P3 OR P4 OR P5 OR P6 OR P7 OR P8 OR P9 OR P10 OR P11 OR P12 OR P13 OR P14 OR P15 OR P16 OR P17 OR P18 OR P19 OR P20

Women

WO1	Woman
WO2	Women
WO3	Female*
WO4	Mother*
WO5	maternal
WO6	WO1 OR WO2 OR WO3 OR WO4 OR WO5

Chronic or End-stage kidney disease

K1	Chronic
K2	Long-term
K3	Long term
K4	End-stage
K5	End stage
K6	K1 OR K2 OR K3 OR K4 OR K5
K7	Kidney*
K8	renal
K9	K7 OR K8
K10	Insufficien*
K11	Disease*
K12	Fail*
K13	Impair*
K14	Dysfunction*
K15	K10 OR K11 OR K12 OR K13 OR K14
K16	K6 AND K9 AND K15
K17	CKD
K18	CRD
K19	CKF
K20	CRF
K21	ESKD
K22	ESRD
K23	ESKF
K24	ESRF
K25	K17 OR K18 OR K19 OR K20 OR K21 OR K22 OR K23 OR K24
K26	K16 OR K25

Search strategy

(H30 OR D10 OR W24 OR P21) AND WO6 AND K26
