# Supplementary material for: Adverse pregnancy outcomes and long-term risk of maternal renal disease: a systematic review and meta-analysis protocol
Source: BMJ Open. 2019 May 5;9(5):e027180. doi: 10.1136/bmjopen-2018-027180 (PMC6502020; doi:10.1136/bmjopen-2018-027180)
Supplement: Author's manuscript [file bmjopen-2018-027180.draft_revisions.pdf]

BMJ Open is committed to open peer review. As part of this commitment we make the peer review history of every article we publish publicly available.

When an article is published we post the peer reviewers' comments and the authors' responses online. We also post the versions of the paper that were used during peer review. These are the versions that the peer review comments apply to.

The versions of the paper that follow are the versions that were submitted during the peer review process. They are not the versions of record or the final published versions. They should not be cited or distributed as the published version of this manuscript.

BMJ Open is an open access journal and the full, final, typeset and author-corrected version of record of the manuscript is available on our site with no access controls, subscription charges or pay-per-view fees (http://bmjopen.bmj.com).

If you have any questions on BMJ Open's open peer review process please email info.bmjopen@bmj.com

BMJ Open

ADVERSE PREGNANCY OUTCOMES AND LONG-TERM RISK OF MATERNAL RENAL DISEASE: A SYSTEMATIC REVIEW AND META-ANALYSIS PROTOCOL.

Journal:	*BMJ Open*
Manuscript ID	bmjopen-2018-027180
Article Type:	Protocol
Date Submitted by the Author:	09-Oct-2018
Complete List of Authors:	Barrett, Peter; University College Cork, School of Public Health; University College Cork National University of Ireland, Irish Centre for Fetal and Neonatal Translational Research<br>McCarthy, Fergus; The Irish Centre for Fetal and Neonatal Translational Research (INFANT), Department of Obstetrics and Gynecology<br>Kublickiene, Karolina; Karolinska institutet Department of Clinical Sciences Intervention and Technology,<br>Evans, Marie; Karolinska institutet Department of Clinical Sciences Intervention and Technology<br>Cormican, Sarah; Galway University Hospital, Department of Nephrology<br>Judge, Conor; Galway University Hospital, Department of Nephrology<br>Perry, Ivan; University College Cork, Epidemiology and Public Health<br>Kublickas, Marius; Karolinska institutet Department of Clinical Sciences Intervention and Technology<br>Stenvinkel, Peter; Karolinska institutet Department of Clinical Sciences Intervention and Technology<br>Khashan, Ali; University College Cork, Department of Epidemiology and Public Health
Keywords:	Chronic renal failure < NEPHROLOGY, End stage renal failure < NEPHROLOGY, Diabetes in pregnancy < DIABETES & ENDOCRINOLOGY, OBSTETRICS, EPIDEMIOLOGY

SCHOLARONE™
Manuscripts

ADVERSE PREGNANCY OUTCOMES AND LONG-TERM RISK OF MATERNAL RENAL DISEASE: A SYSTEMATIC REVIEW AND META-ANALYSIS PROTOCOL.

Peter Barrett[1,2], Fergus McCarthy[2], Karolina Kublickiene[3], Marie Evans[3], Sarah Cormican[4,5], Conor Judge[4], Ivan J Perry[1], Marius Kublickas[3], Peter Stenvinkel[3], Ali Khashan[1,2].

Corresponding author: Dr. Peter Barrett; peter.barrett@ucc.ie; +353 86 3773594; School of Public Health, Floor 4 Western Gateway Building, University College Cork, Cork City, Ireland.

Author Affiliations:

1: School of Public Health, Western Gateway Building, University College Cork, Cork, Ireland.

2: The Irish Centre for Fetal and Neonatal Translational Research (INFANT), Cork University Maternity Hospital & University College Cork, Cork, Ireland

3: Department of Clinical Science, Intervention and Technology (CLINTEC), Karolinska Universitetssjukhuset, Stockholm, Sweden.

4: Department of Nephrology, University Hospital Galway, Galway, Ireland.

5: Regenerative Medicine Institute (REMEDI), School of Medicine, National University of Ireland, Galway, Ireland

Word count: 2541, excluding title page, abstract, references.

For peer review only

For peer review only - http://bmjopen.bmj.com/site/about/guidelines.xhtml

ABSTRACT

Introduction:

Adverse pregnancy outcomes, such as hypertensive disorders of pregnancy (HDP), gestational diabetes (GDM), and preterm birth have been linked to maternal cardiovascular disease in later life. Preeclampsia (PE) is associated with an increased risk of postpartum microalbuminuria, but there is no clear consensus on whether HDP increase the risk of maternal chronic kidney disease (CKD) and end-stage kidney disease (ESKD). Similarly, it is uncertain whether GDM, preterm birth, and delivery of low birth weight infants independently predict the risk of maternal renal disease in later life. The aims of this proposed systematic review and meta-analysis are to summarise the available evidence examining the association between adverse outcomes of pregnancy (HDP, GDM, preterm birth, delivery of low birth weight infant) and later maternal renal disease, and to synthesise the results of relevant studies.

Methods and analysis:

A systematic search of PubMed, EMBASE and Web of Science will be undertaken using a detailed pre-specified search strategy. Two authors will independently review the titles and abstracts of all studies, perform data extraction, and appraise the quality of included studies using a bias classification tool. Original case-control and cohort studies published in English will be considered for inclusion. Primary outcomes of interest will be CKD and ESKD; secondary outcomes will be hospitalisation for renal disease and deaths from renal disease. Meta-analyses will be performed to calculate overall pooled estimates using the generic inverse variance method. The systematic review will follow the Meta-analyses Of Observational Studies in Epidemiology (MOOSE) guidelines.

Ethics and dissemination:

This systematic review and meta-analysis will be based on published data, and thus there is no requirement for ethics approval. The results will be shared through publication in a peer-reviewed journal, and through presentations at academic conferences.

Registration:

CRD42018110891

Keywords: preeclampsia; birth weight; gestational diabetes; preterm birth; hypertension; pregnancy; chronic kidney disease; end-stage kidney disease;

For peer review only

For peer review only - http://bmjopen.bmj.com/site/about/guidelines.xhtml

STRENGTHS & LIMITATIONS OF THIS STUDY

- This proposed systematic review and meta-analysis will provide updated knowledge on the associations between common adverse pregnancy outcomes (i.e. hypertensive disorders of pregnancy, gestational diabetes, preterm birth, delivery of low birth weight infants) and the long-term maternal risk of renal disease.
- The high prevalence of adverse pregnancy outcomes, and of chronic kidney disease, suggest that any potential associations would have important public health implications
- Screening for eligible studies, data extraction, and quality assessment will be undertaken by two independent reviewers to minimise the potential for reviewer bias.
- A considerable degree of heterogeneity is anticipated between studies due to differences in diagnostic methods for both exposures and outcomes, and varying lengths of follow-up.
- Only published studies in the English language will be included.

For peer review only

INTRODUCTION

Reproductive history is increasingly recognised as an important predictor of later health outcomes in women, as the "stress test" of pregnancy can predispose to chronic disease. Complications of pregnancy have been strongly linked with later cardiovascular disease (CVD). Preeclampsia (PE), gestational hypertension, and gestational diabetes (GDM) are now recognised as independent cardiovascular risk factors (1, 2), while preterm birth, growth restriction, and gestational weight gain have also been linked to future CVD (3-5). By comparison, less is known about the long-term risk of maternal renal disease following complications of pregnancy.

There is some evidence to suggest that PE increases the risk of maternal kidney disease later in life. A meta-analysis of seven small prospective studies identified an increased risk of microalbuminuria among women who experienced PE compared with those who had normal pregnancies after a mean of seven years follow-up (6). A large Norwegian cohort study suggested that preeclamptic women were at long-term risk of end-stage kidney disease (ESKD) (7). However, the proposed association may be complicated by the strong links between PE and unmeasured cardiovascular risk factors, such as maternal obesity, which can independently increase the risk of chronic kidney disease (CKD) (8).

The long-term risk of CKD among these women has not been comprehensively addressed by studies to date. It is biologically plausible that PE predisposes women to higher risk of CKD; PE is associated with abnormal placental development, and subsequent development of generalised maternal endothelial dysfunction (9). PE may thus result in lasting renal endothelial damage, which may increase maternal risk of CKD and ESKD (10). PE is just one of several hypertensive disorders of pregnancy (HDP), and it is unknown if women who experience gestational hypertension, or PE superimposed on chronic hypertension are also at increased long-term risk of maternal kidney disease.

A range of other complications of pregnancy may increase the risk of future kidney disease in women. Low birth weight and preterm delivery have been reported to increase the risk of ESKD in women with PE (7). However, it is uncertain whether these associations persist independently, or whether birth weight and gestational age are mediators of the association with PE. In one Norwegian cohort study, preterm birth was reported to independently increase the risk of ESKD, but this study was restricted to women with pre-existing diabetes and is not generalisable to the wider population (11).

GDM is an established risk factor for type 2 diabetes (12), and may thereby increase the risk of diabetic nephropathy. However, GDM is also an independent risk factor for vascular endothelial dysfunction (13, 14), and is a plausible independent risk factor for maternal kidney disease. To date there has been limited research to examine whether GDM predicts CKD risk independent of subsequent type 2 diabetes.

Worldwide, 3-5% of pregnancies are complicated by PE (15); 5-18% of births are preterm (16); 11% have low birth weight (17); and 5-13% are complicated by GDM (18, 19). The global prevalence of CKD is 11-13% (20), and thus, any true associations between these exposures and CKD may have potentially important public health implications, particularly in resource-poor settings where adverse pregnancy outcomes are more prevalent. Women with complications of pregnancy, who may be at risk of CKD, may benefit from future risk reduction interventions, or enhanced community-based follow-up care to mitigate against possible progression to renal disease. Only a minority of individuals with CKD will ever progress to ESKD, requiring dialysis or renal transplant, but they experience premature mortality (21, 22), and place a considerable economic and resource burden on health systems (23).

The aim of this systematic review and meta-analysis is to summarise the available evidence examining the association between adverse pregnancy outcomes and long-term maternal renal

For peer review only - http://bmjopen.bmj.com/site/about/guidelines.xhtml

disease. The adverse outcomes of interest include HDP (including PE, gestational hypertension, chronic hypertension, and PE superimposed on chronic hypertension), preterm birth, delivery of a low birth weight infant, and GDM. The protocol has been registered on PROSPERO (https://www.crd.york.ac.uk/prospero/), registration number CRD42018110891.

Population

Women who have had at least one pregnancy of at least 23 weeks gestation

Exposures

Any one of the following adverse pregnancy outcomes:

1. Diagnosis of HDP (including PE, gestational hypertension, or other HDP)
2. Pre-term birth
3. Diagnosis of GDM
4. Delivery of a low birth weight infant (including infants who were small for gestational age)

For any of these adverse outcomes, they can be defined either using established clinical criteria, hospital records, or self-reporting of a doctor diagnosis.

Comparison

Women who never had a corresponding adverse outcome in pregnancy. For example, women who experienced a preterm birth in at least one pregnancy will be compared with women who never experienced a preterm birth.

Outcomes

Primary outcome 1: CKD

Primary outcome 2: ESKD

These primary outcomes can be defined either using established clinical criteria, or hospital records.

Secondary outcomes: (1) Hospitalisation for renal disease, (2) Deaths from renal disease

For peer review only

Review question

Does the presence of an adverse pregnancy outcome (i.e. HDP, preterm birth, delivery of a low birth weight infant, or GDM) increase the risk of maternal kidney disease in later life?

METHODS AND DESIGN

This protocol was drafted using the Preferred Reporting Items for Systematic Reviews and Meta-Analysis Protocols (PRISMA-P) checklist (24). The proposed systematic review and meta-analysis will follow the Meta-analyses Of Observational Studies in Epidemiology (MOOSE) guidelines (25).

For peer review only

Search strategy

The lead author (PB) will undertake a systematic search using the following databases: PubMed, EMBASE and Web of Science. Peer-reviewed journal articles published in English language, from inception of databases to 31 July 2018, will be included. A detailed search strategy has been compiled, and the search terms will be combined using Boolean Logic where appropriate (AND, OR). The detailed search strategy is available in Appendix 1.

Searches of the electronic databases will be supplemented by hand-searching all included papers to identify any further potentially relevant studies.

Study selection

The titles and abstracts of studies retrieved from each database search will be stored in EndNote reference manager, and de-duplicated. Two review authors (PB, SC) will screen all titles and abstracts for potentially relevant studies. The full text of relevant studies will then be retrieved and will be screened for compliance with eligibility criteria by two reviewers (PB, CJ). If consensus on eligibility cannot be achieved, a third review author will be consulted (AK). For any articles which do not meet inclusion criteria, the reasons for rejection will be noted. A MOOSE flow diagram documenting the process of study selection will be completed.

Inclusion criteria

- Case-control studies and cohort studies (prospective or retrospective).
- Data provided on an adverse pregnancy outcome of interest (i.e. diagnosis of HDP, preterm birth, low birth weight, or GDM) as an exposure variable.
- Data provided on a diagnosis of either CKD or ESKD as an outcome variable.
- Provides a measure of association between one or more of the adverse pregnancy outcomes and CKD or ESKD.
- The diagnosis of maternal CKD or ESKD is made at least three months after pregnancy has ended.

For peer review only - http://bmjopen.bmj.com/site/about/guidelines.xhtml

- Data must be from an original study
- Only English-language studies will be considered, including all years from inception of the electronic databases until July 2018.
- Peer-reviewed literature only will be included.

Exclusion criteria

- Non-human studies.
- Studies that are not in English.
- Studies focused on CKD/ESKD risk in the offspring.
- Studies focused on risk of maternal acute kidney injury, without reference to subsequent CKD/ESKD.
- Case reports, case series, letters, commentaries, notes, and editorials.
- Studies focused on women with pre-existing renal disease.

Data extraction

Data from all eligible studies will be extracted by two reviewers (PB, SC) using a standardised data collection form, including the author and year of publication, the study design, the exposure(s) and outcome(s) of interest, the definition used for each exposure and outcome, the stage/severity of the outcome (i.e. CKD), length of follow-up, the sample size, the confounders adjusted for (if any) and the crude and adjusted measures of association. Where necessary, corresponding authors of published studies will be contacted to obtain any information needed relating to effect estimates. Where effect estimates are not available, absolute numbers of events will be extracted, and crude measures of association will be calculated. If discrepancies arise in data extraction, these will be discussed between reviewers, and where necessary, a third reviewer will be consulted to achieve consensus (AK).

Quality appraisal of included studies

The quality of all included studies will be independently assessed by two reviewers using an established quality assessment tool for observational studies (PB, CJ). This tool has been described in detail elsewhere (26). Six types of bias will be assessed: selection, exposure, outcome, analytical, attrition, and confounding. For each study, each component will be assigned a risk of bias category: minimal, low, moderate, high, or not reported. For example, selection bias will be categorised as 'minimal' if the sample was from a 'consecutive unselected population', whereas selection bias will be categorised as 'high' if sample selection is unclear and if the sample is not representative of the population of interest. For each included study, the overall likelihood of bias will be assessed and

For peer review only - http://bmjopen.bmj.com/site/about/guidelines.xhtml

reported. Where discrepancies in quality appraisal arise, a third reviewer will be consulted to achieve consensus (AK).

Data synthesis and assessment for heterogeneity

Separate meta-analyses will be undertaken for each of the exposure variables and two primary outcomes where possible. Each meta-analysis will be undertaken to calculate the pooled estimate of the relationship between the adverse outcome of interest and subsequent development of maternal CKD or ESKD. For example, for preterm birth as the adverse outcome of interest, a meta-analysis will be undertaken to investigate the association between (1) preterm birth and CKD and (2) preterm birth and ESKD. For HDP, a meta-analysis will be undertaken to investigate the association between (1) any HDP and CKD and (2) any HDP and ESKD. Where possible, subgroup analyses will investigate the associations between PE, gestational hypertension, and any other HDP with each of the primary outcomes respectively. For delivery of infants with low birth weight, a meta-analysis will be undertaken to investigate the association between (1) low birth weight infant and maternal CKD and (2) low birth weight infant and maternal ESKD. Where possible, subgroup analyses will investigate the associations between small for gestational age infants with each of the primary outcomes respectively.

Both crude and adjusted effect estimates will be displayed using the generic inverse variance method. Adjustment will be based on the definition outlined in each of the individual studies. Heterogeneity will be explored based on I^2 values and chi-squared statistics. Random-effects models will be used if moderate or high levels of heterogeneity are observed between the studies of interest. If studies cannot be meaningfully combined in a meta-analysis, they will be presented in tabular format.

We will perform the following subgroup/sensitivity analyses where the data allow, using RevMan 5.3: (1) study type (case-control vs. cohort study), (2) stage/severity of CKD, (3) ethnic group, (4) length of follow-up after index pregnancy, (5) number of pregnancies affected by the adverse outcome, (6) measurement of exposure and outcome data (self-reported vs medical records vs laboratory measurements), (7) study quality (minimal/low risk of bias vs. moderate/high risk of bias. Furthermore, any studies which have not excluded women with pre-existing renal disease, or have not adjusted for this factor, will be considered in a separate subgroup analysis.

Where 10 or more studies are included in a meta-analysis, we will assess for publication bias. The trim and fill method will be used to identify and correct for funnel plot asymmetry arising from publication bias if appropriate (27).

Ethics and dissemination

This protocol is based on published data, and thus there is no requirement for ethics approval. The results will be disseminated through publication in a peer-reviewed journal, and through presentations at academic conferences.

For peer review only - http://bmjopen.bmj.com/site/about/guidelines.xhtml

Potential limitations

There are a number of limitations anticipated in this review. Publication bias may reduce the likelihood of retrieving studies which report non-significant associations between adverse pregnancy outcomes and maternal renal disease. Due to limited resources, only studies which are published in the English language will be included. Publications which only use biomarkers of renal function as outcome variables (e.g. microalbuminuria, albumin/creatinine ratio, estimated glomerular filtration rate) will not be included unless they are directly related to a diagnosis of CKD or ESKD.

A degree of heterogeneity is anticipated between studies. Differences in diagnostic methods are likely for both exposures and outcomes. The timeframe for follow-up is likely to vary considerably between studies, with those women followed up for longer more likely to have developed renal disease. Many individuals with early CKD are asymptomatic, and may not be aware of their diagnosis unless engaged in screening programmes, whereas those with ESKD are typically aware of their diagnosis due to their need for dialysis or renal transplant. Differences in sampling frames are also likely to lead to heterogeneity. Thus, a random effects model will be used for meta-analyses with moderate or high heterogeneity.

The presence of selection bias and residual confounding is a concern in all observational studies. Potential confounders may include maternal age, ethnic group, socio-economic status, parity, hypertension, diabetes, cardiovascular disease, systemic inflammatory disease, hyperlipidaemia, obesity, smoking. Our meta-analyses will display both crude and adjusted results where possible, basing adjustment on the definition outlined in each individual study. Where possible, a subgroup analysis will be undertaken to examine effect estimates which are adjusted more fully for these confounders.

CONCLUSIONS

There is a lack of consensus on whether adverse pregnancy outcomes, such as HDP, preterm birth, delivery of a low birth-weight infant, and GDM, independently increase the risk of maternal CKD and ESKD. This systematic review and meta-analysis will summarise the available evidence which has examined these associations, thus providing novel information on the role of pregnancy-related factors in the aetiology of maternal renal disease.

ACKNOWLEDGEMENTS

Competing interests

None declared

For peer review only - http://bmjopen.bmj.com/site/about/guidelines.xhtml

Funding

This work was performed within the Irish Clinical Academic Training (ICAT) Programme, supported by the Wellcome Trust and the Health Research Board (Grant Number 203930/B/16/Z), the Health Service Executive National Doctors Training and Planning and the Health and Social Care, Research and Development Division, Northern Ireland.

Author contributions

PB, FMcC, IP and AK conceived and designed the protocol, and PB drafted the protocol manuscript. PB developed the search strategy, with input from FMcC, IP and AK. PB, CJ and AK planned the data extraction. PB, SC and AK planned the quality appraisal of all included studies. PB, FMcC, IP, SC, CJ, KK, MK, ME and AK critically revised the manuscript for methodological and intellectual content. All authors approved the final version.

For peer review only

REFERENCES

1. Mosca L, Benjamin EJ, Berra K, Bezanson JL, Dolor RJ, Lloyd-Jones DM, et al. Effectiveness-based guidelines for the prevention of cardiovascular disease in women--2011 update: a guideline from the American Heart Association. J Am Coll Cardiol. 2011;57(12):1404-23.
2. Bushnell C, McCullough L. Stroke prevention in women: synopsis of the 2014 American Heart Association/American Stroke Association guideline. Ann Intern Med. 2014;160(12):853-7.
3. van Kesteren F, Visser S, Hermes W, Teunissen PW, Franx A, van Pampus MG, et al. Counselling and management of cardiovascular risk factors after preeclampsia. Hypertens Pregnancy. 2016;35(1):55-61.
4. Cirillo PM, Cohn BA. Pregnancy complications and cardiovascular disease death: 50-year follow-up of the Child Health and Development Studies pregnancy cohort. Circulation. 2015;132(13):1234-42.
5. Heida KY, Velthuis BK, Oudijk MA, Reitsma JB, Bots ML, Franx A, et al. Cardiovascular disease risk in women with a history of spontaneous preterm delivery: A systematic review and meta-analysis. Eur J Prev Cardiol. 2016;23(3):253-63.
6. McDonald S, Han Z, Michael W, Gerstein H, Devereaux P. Renal disease after preeclampsia: A systematic review and meta-analyses. Pregnancy Hypertension. 2010;1:S41.
7. Vikse BE, Irgens LM, Leivestad T, Skjaerven R, Iversen BM. Preeclampsia and the risk of end-stage renal disease. N Engl J Med. 2008;359(8):800-9.
8. Lastra G, Manrique C, Sowers JR. Obesity, cardiometabolic syndrome, and chronic kidney disease: the weight of the evidence. Adv Chronic Kidney Dis. 2006;13(4):365-73.
9. Zhou Y, Damsky CH, Fisher SJ. Preeclampsia is associated with failure of human cytotrophoblasts to mimic a vascular adhesion phenotype. One cause of defective endovascular invasion in this syndrome? J Clin Invest. 1997;99(9):2152-64.
10. Rangaswami J, Naranjo M, McCullough PA. Preeclampsia as a Form of Type 5 Cardiorenal Syndrome: An Underrecognized Entity in Women's Cardiovascular Health. Cardiorenal Med. 2018;8(2):160-72.
11. Sandvik MK, Iversen BM, Irgens LM, Skjaerven R, Leivestad T, Softeland E, et al. Are adverse pregnancy outcomes risk factors for development of end-stage renal disease in women with diabetes? Nephrol Dial Transplant. 2010;25(11):3600-7.
12. Bellamy L, Casas JP, Hingorani AD, Williams D. Type 2 diabetes mellitus after gestational diabetes: a systematic review and meta-analysis. Lancet. 2009;373(9677):1773-9.
13. Knock GA, McCarthy AL, Lowy C, Poston L. Association of gestational diabetes with abnormal maternal vascular endothelial function. Br J Obstet Gynaecol. 1997;104(2):229-34.
14. Lappas M. Markers of endothelial cell dysfunction are increased in human omental adipose tissue from women with pre-existing maternal obesity and gestational diabetes. Metabolism. 2014;63(6):860-73.
15. Hypertension in pregnancy. Report of the American College of Obstetricians and Gynecologists' Task Force on Hypertension in Pregnancy. Obstet Gynecol. 2013;122(5):1122-31.
16. Blencowe H, Cousens S, Oestergaard MZ, Chou D, Moller AB, Narwal R, et al. National, regional, and worldwide estimates of preterm birth rates in the year 2010 with time trends since 1990 for selected countries: a systematic analysis and implications. Lancet. 2012;379(9832):2162-72.
17. The World Bank. Low-birthweight babies (% of births) 2018 [Available from: https://data.worldbank.org/indicator/SH.STA.BRTW.ZS.
18. Eades CE, Cameron DM, Evans JMM. Prevalence of gestational diabetes mellitus in Europe: A meta-analysis. Diabetes Res Clin Pract. 2017;129:173-81.
19. Zhu Y, Zhang C. Prevalence of Gestational Diabetes and Risk of Progression to Type 2 Diabetes: a Global Perspective. Curr Diab Rep. 2016;16(1):7.
20. Hill NR, Fatoba ST, Oke JL, Hirst JA, O'Callaghan CA, Lasserson DS, et al. Global Prevalence of Chronic Kidney Disease - A Systematic Review and Meta-Analysis. PLoS One. 2016;11(7):e0158765.
21. Tonelli M, Wiebe N, Culleton B, House A, Rabbat C, Fok M, et al. Chronic kidney disease and mortality risk: a systematic review. J Am Soc Nephrol. 2006;17(7):2034-47.
22. Di Angelantonio E, Chowdhury R, Sarwar N, Aspelund T, Danesh J, Gudnason V. Chronic kidney disease and risk of major cardiovascular disease and non-vascular mortality: prospective population based cohort study. BMJ. 2010;341:c4986.
23. Levin A, Tonelli M, Bonventre J, Coresh J, Donner JA, Fogo AB, et al. Global kidney health 2017 and beyond: a roadmap for closing gaps in care, research, and policy. Lancet. 2017;390(10105):1888-917.
24. Moher D, Shamseer L, Clarke M, Ghersi D, Liberati A, Petticrew M, et al. Preferred reporting items for systematic review and meta-analysis protocols (PRISMA-P) 2015 statement. Syst Rev. 2015;4:1.
25. Stroup DF, Berlin JA, Morton SC, Olkin I, Williamson GD, Rennie D, et al. Meta-analysis of observational studies in epidemiology: a proposal for reporting. Meta-analysis Of Observational Studies in Epidemiology (MOOSE) group. JAMA. 2000;283(15):2008-12.

For peer review only - http://bmjopen.bmj.com/site/about/guidelines.xhtml

26. McDonald SD, Han Z, Mulla S, Murphy KE, Beyene J, Ohlsson A. Preterm birth and low birth weight among in vitro fertilization singletons: a systematic review and meta-analyses. Eur J Obstet Gynecol Reprod Biol. 2009;146(2):138-48.
27. Weinhandl ED, Duval S. Generalization of trim and fill for application in meta-regression. Res Synth Methods. 2012;3(1):51-67.

For peer review only

For peer review only - http://bmjopen.bmj.com/site/about/guidelines.xhtml

Appendix 1 – Search Strategy

Preeclampsia & gestational hypertension

H1	Antenatal
H2	Ante-natal
H3	Prenatal
H4	Pre-natal
H5	Pregnan*
H6	Gestation*
H7	H1 OR H2 OR H3 OR H4 OR H5 OR H6
H8	Pre Eclampsia
H9	Preeclampsia
H10	Pre-eclampsia
H11	Eclampsia
H12	Edema-Proteinuria-Hypertension
H13	Edema Proteinuria Hypertension
H14	Oedema-Proteinuria-Hypertension
H15	Oedema Proteinuria Hypertension
H16	EPH
H17	Toxemia*
H18	Toxaemia*
H19	Gestosis
H20	(H19 OR H7)
H21	Toxemia* OR Toxaemia* or EPH
H22	H21 AND H20
H23	Gestational hypertension
H24	Pregnancy-induced hypertension
H25	Pregnancy induced hypertension
H26	Hypertensi*
H27	Blood pressure
H28	H26 OR H27
H29	H28 AND H7
H30	H8 OR H9 OR H10 OR H11 OR H12 OR H13 OR H14 OR H15 OR H22 OR H23 OR H24 OR H25 OR H29

Gestational diabetes

D1	Pregnancy-Induced diabetes
D2	Pregnancy Induced diabetes
D3	Gestational Diabetes
D4	diabetes in pregnancy
D5	diabetes of pregnancy
D6	Diabetes mellitus in pregnancy
D7	Diabetes mellitus of pregnancy
D8	pregnancy-related diabetes
D9	pregnancy related diabetes
D10	D1 OR D2 OR D3 OR D4 OR D5 OR D6 OR D7 OR D8 OR D9 OR D10

For peer review only - http://bmjopen.bmj.com/site/about/guidelines.xhtml

Low birth weight

W1	Low Birth Weight
W2	Low-Birth-Weight
W3	Low Birth Weights
W4	Low-Birth-Weights
W5	Low Birthweight
W6	Low Birthweights
W7	Extremely Low Birth Weight
W8	Extremely-Low-Birth-Weight
W9	Extremely-Low-Birth-Weights
W10	Extremely Low Birthweight
W11	Very Low Birth Weight
W12	Very-Low-Birth-Weight
W13	Very-Low-Birth-Weights
W14	Very Low Birthweight
W15	Birth Weights
W16	Birth Weight
W17	Birthweight
W18	LBW
W19	VLBW
W20	ELBW
W21	Small for gestational age
W22	Growth restrict*
W23	IUGR
W24	W1 OR W2 OR W3 OR W4 OR W5 OR W6 OR W6 OR W7 OR W8 OR W9 OR W10 OR W11 OR W12 OR W13 OR W14 OR W15 OR W16 OR W17 OR W18 OR W19 OR W20 OR W21 OR W22 OR W23

Preterm birth or SGA

P1	Premature Birth
P2	Preterm Birth
P3	Premature Births
P4	Preterm Births
P5	Pre-term Birth
P6	Pre-term Births
P7	Gestational age
P8	Gestational ages
P9	Fetal Age
P10	Foetal Age
P11	Fetal Ages
P12	Foetal Ages
P13	Premature Labor
P14	Preterm Labor
P15	Pre-term Labor
P16	Premature Labour
P17	Preterm Labour
P18	Pre-term Labour
P19	Premature Obstetric Labor
P20	Premature Obstetric Labour
P21	P1 OR P2 OR P3 OR P4 OR P5 OR P6 OR P7 OR P8 OR P9 OR P10 OR P11 OR P12 OR P13 OR P14 OR P15 OR P16 OR P17 OR P18 OR P19 OR P20

For peer review only - http://bmjopen.bmj.com/site/about/guidelines.xhtml

Women

WO1	Woman
WO2	Women
WO3	Female*
WO4	Mother*
WO5	maternal
WO6	WO1 OR WO2 OR WO3 OR WO4 OR WO5

Chronic or End-stage kidney disease

K1	Chronic
K2	Long-term
K3	Long term
K4	End-stage
K5	End stage
K6	K1 OR K2 OR K3 OR K4 OR K5
K7	Kidney*
K8	renal
K9	K7 OR K8
K10	Insufficien*
K11	Disease*
K12	Fail*
K13	Impair*
K14	Dysfunction*
K15	K10 OR K11 OR K12 OR K13 OR K14
K16	K6 AND K9 AND K15
K17	CKD
K18	CRD
K19	CKF
K20	CRF
K21	ESKD
K22	ESRD
K23	ESKF
K24	ESRF
K25	K17 OR K18 OR K19 OR K20 OR K21 OR K22 OR K23 OR K24
K26	K16 OR K25

Search strategy

(H30 OR D10 OR W24 OR P21) AND WO6 AND K26

For peer review only - http://bmjopen.bmj.com/site/about/guidelines.xhtml

PRISMA-P (Preferred Reporting Items for Systematic review and Meta-Analysis Protocols) 2015 checklist: recommended items to address in a systematic review protocol*

Section and topic	Item No	Checklist item	(Page No.#)
**ADMINISTRATIVE INFORMATION**			
Title:			
Identification	1a	Identify the report as a protocol of a systematic review	1
Update	1b	If the protocol is for an update of a previous systematic review, identify as such	n/a
Registration	2	If registered, provide the name of the registry (such as PROSPERO) and registration number	5
Authors:			
Contact	3a	Provide name, institutional affiliation, e-mail address of all protocol authors; provide physical mailing address of corresponding author	1
Contributions	3b	Describe contributions of protocol authors and identify the guarantor of the review	10
Amendments	4	If the protocol represents an amendment of a previously completed or published protocol, identify as such and list changes; otherwise, state plan for documenting important protocol amendments	n/a
Support:			
Sources	5a	Indicate sources of financial or other support for the review	10
Sponsor	5b	Provide name for the review funder and/or sponsor	10
Role of sponsor or funder	5c	Describe roles of funder(s), sponsor(s), and/or institution(s), if any, in developing the protocol	10
**INTRODUCTION**			
Rationale	6	Describe the rationale for the review in the context of what is already known	4
Objectives	7	Provide an explicit statement of the question(s) the review will address with reference to participants, interventions, comparators, and outcomes (PICO)	4, 5
**METHODS**			
Eligibility criteria	8	Specify the study characteristics (such as PICO, study design, setting, time frame) and report characteristics (such as years considered, language, publication status) to be used as criteria for eligibility for the review	5, 6, 7
Information sources	9	Describe all intended information sources (such as electronic databases, contact with study authors, trial registers or other grey literature sources) with planned dates of coverage	6, 7
Search strategy	10	Present draft of search strategy to be used for at least one electronic database, including planned limits, such that it could be repeated	13-15

For peer review only

Study records:			
Data management	11a	Describe the mechanism(s) that will be used to manage records and data throughout the review	6
Selection process	11b	State the process that will be used for selecting studies (such as two independent reviewers) through each phase of the review (that is, screening, eligibility and inclusion in meta-analysis)	6
Data collection process	11c	Describe planned method of extracting data from reports (such as piloting forms, done independently, in duplicate), any processes for obtaining and confirming data from investigators	7
Data items	12	List and define all variables for which data will be sought (such as PICO items, funding sources), any pre-planned data assumptions and simplifications	7
Outcomes and prioritization	13	List and define all outcomes for which data will be sought, including prioritization of main and additional outcomes, with rationale	5
Risk of bias in individual studies	14	Describe anticipated methods for assessing risk of bias of individual studies, including whether this will be done at the outcome or study level, or both; state how this information will be used in data synthesis	7, 8
Data synthesis	15a	Describe criteria under which study data will be quantitatively synthesised	8
	15b	If data are appropriate for quantitative synthesis, describe planned summary measures, methods of handling data and methods of combining data from studies, including any planned exploration of consistency (such as I^2, Kendall's τ)	8
	15c	Describe any proposed additional analyses (such as sensitivity or subgroup analyses, meta-regression)	8
	15d	If quantitative synthesis is not appropriate, describe the type of summary planned	8
Meta-bias(es)	16	Specify any planned assessment of meta-bias(es) (such as publication bias across studies, selective reporting within studies)	8
Confidence in cumulative evidence	17	Describe how the strength of the body of evidence will be assessed (such as GRADE)<br><br>***Only observational studies will be included. Strength of evidence will be based on quality assessment and adjustment for confounders. If possible, a hierarchy of adjustment will be used in sensitivity analysis*	7, 8**

*** It is strongly recommended that this checklist be read in conjunction with the PRISMA-P Explanation and Elaboration (cite when available) for important clarification on the items. Amendments to a review protocol should be tracked and dated. The copyright for PRISMA-P (including checklist) is held by the PRISMA-P Group and is distributed under a Creative Commons Attribution Licence 4.0.**

From: Shamseer L, Moher D, Clarke M, Ghersi D, Liberati A, Petticrew M, Shekelle P, Stewart L, PRISMA-P Group. Preferred reporting items for systematic review and meta-analysis protocols (PRISMA-P) 2015: elaboration and explanation. BMJ. 2015 Jan 2;349(jan02 1):g7647.

For peer review only

BMJ Open

ADVERSE PREGNANCY OUTCOMES AND LONG-TERM RISK OF MATERNAL RENAL DISEASE: A SYSTEMATIC REVIEW AND META-ANALYSIS PROTOCOL.

Journal:	*BMJ Open*
Manuscript ID	bmjopen-2018-027180.R1
Article Type:	Protocol
Date Submitted by the Author:	25-Feb-2019
Complete List of Authors:	Barrett, Peter; University College Cork, School of Public Health; University College Cork National University of Ireland, Irish Centre for Fetal and Neonatal Translational Research<br>McCarthy, Fergus; The Irish Centre for Fetal and Neonatal Translational Research (INFANT), Department of Obstetrics and Gynecology<br>Kublickiene, Karolina; Karolinska institutet Department of Clinical Sciences Intervention and Technology,<br>Evans, Marie; Karolinska institutet Department of Clinical Sciences Intervention and Technology<br>Cormican, Sarah; Galway University Hospital, Department of Nephrology<br>Judge, Conor; Galway University Hospital, Department of Nephrology<br>Perry, Ivan; University College Cork, Epidemiology and Public Health<br>Kublickas, Marius; Karolinska institutet Department of Clinical Sciences Intervention and Technology<br>Stenvinkel, Peter; Karolinska institutet Department of Clinical Sciences Intervention and Technology<br>Khashan, Ali; University College Cork, Department of Epidemiology and Public Health
**Primary Subject Heading**:	Epidemiology
Secondary Subject Heading:	Obstetrics and gynaecology, Renal medicine
Keywords:	Chronic renal failure < NEPHROLOGY, End stage renal failure < NEPHROLOGY, Diabetes in pregnancy < DIABETES & ENDOCRINOLOGY, OBSTETRICS, EPIDEMIOLOGY

SCHOLARONE™
Manuscripts

ADVERSE PREGNANCY OUTCOMES AND LONG-TERM RISK OF MATERNAL RENAL DISEASE: A SYSTEMATIC REVIEW AND META-ANALYSIS PROTOCOL.

Peter Barrett[1,2], Fergus McCarthy[2], Karolina Kublickiene[3], Marie Evans[3], Sarah Cormican[4,5], Conor Judge[4], Ivan J Perry[1], Marius Kublickas[3], Peter Stenvinkel[3], Ali S Khashan[1,2].

Corresponding author: Dr. Peter Barrett; peterbarrett1@hotmail.com; +353 86 3773594; School of Public Health, Floor 4 Western Gateway Building, University College Cork, Cork City, Ireland.

Author Affiliations:

1: School of Public Health, Western Gateway Building, University College Cork, Cork, Ireland.

2: The Irish Centre for Fetal and Neonatal Translational Research (INFANT), Cork University Maternity Hospital & University College Cork, Cork, Ireland

3: Department of Clinical Science, Intervention and Technology (CLINTEC), Karolinska Universitetssjukhuset, Stockholm, Sweden.

4: Department of Nephrology, University Hospital Galway, Galway, Ireland.

5: Regenerative Medicine Institute (REMEDI), School of Medicine, National University of Ireland, Galway, Ireland

Word count: 2541, excluding title page, abstract, references.

For peer review only

For peer review only - http://bmjopen.bmj.com/site/about/guidelines.xhtml

ABSTRACT

Introduction:

Adverse pregnancy outcomes, such as hypertensive disorders of pregnancy (HDP), gestational diabetes (GDM), and preterm birth have been linked to maternal cardiovascular disease in later life. Preeclampsia (PE) is associated with an increased risk of postpartum microalbuminuria, but there is no clear consensus on whether HDP increase the risk of maternal chronic kidney disease (CKD) and end-stage kidney disease (ESKD). Similarly, it is uncertain whether GDM, preterm birth, and delivery of low birth weight infants independently predict the risk of maternal renal disease in later life. The aims of this proposed systematic review and meta-analysis are to summarise the available evidence examining the association between adverse outcomes of pregnancy (HDP, GDM, preterm birth, delivery of low birth weight infant) and later maternal renal disease, and to synthesise the results of relevant studies.

Methods and analysis:

A systematic search of PubMed, EMBASE and Web of Science will be undertaken using a detailed pre-specified search strategy. Two authors will independently review the titles and abstracts of all studies, perform data extraction, and appraise the quality of included studies using a bias classification tool. Original case-control and cohort studies published in English will be considered for inclusion. Primary outcomes of interest will be CKD and ESKD; secondary outcomes will be hospitalisation for renal disease and deaths from renal disease. Meta-analyses will be performed to calculate overall pooled estimates using the generic inverse variance method. The systematic review will follow the Meta-analyses Of Observational Studies in Epidemiology (MOOSE) guidelines.

Ethics and dissemination:

This systematic review and meta-analysis will be based on published data, and thus there is no requirement for ethics approval. The results will be shared through publication in a peer-reviewed journal, and through presentations at academic conferences.

Registration:

CRD42018110891

Keywords: preeclampsia; birth weight; gestational diabetes; preterm birth; hypertension; pregnancy; chronic kidney disease; end-stage kidney disease;

For peer review only

For peer review only - http://bmjopen.bmj.com/site/about/guidelines.xhtml

STRENGTHS & LIMITATIONS OF THIS STUDY

- This proposed systematic review and meta-analysis will provide updated knowledge on the associations between common adverse pregnancy outcomes (i.e. hypertensive disorders of pregnancy, gestational diabetes, preterm birth, delivery of low birth weight infants) and the long-term maternal risk of renal disease.
- The high prevalence of adverse pregnancy outcomes, and of chronic kidney disease, suggest that any potential associations would have important public health implications
- Screening for eligible studies, data extraction, and quality assessment will be undertaken by two independent reviewers to minimise the potential for reviewer bias.
- A considerable degree of heterogeneity is anticipated between studies due to differences in diagnostic methods for both exposures and outcomes, and varying lengths of follow-up.
- Only published studies in the English language will be included.

For peer review only

For peer review only - http://bmjopen.bmj.com/site/about/guidelines.xhtml

INTRODUCTION

Chronic kidney disease (CKD) is a major cause of premature morbidity and mortality worldwide, and its prevalence is estimated at 11-13% among women (1). End-stage kidney disease (ESKD), although relatively rare, causes disproportionately high healthcare burden and expense (2, 3). Globally, rising levels of obesity, metabolic syndrome, and advanced maternal age are resulting in increasing prevalence of adverse pregnancy outcomes, particularly hypertensive disorders of pregnancy (HDP) and gestational diabetes (GDM) (4-6). Preeclampsia (PE), gestational hypertension, and GDM are now recognised as independent cardiovascular risk factors in women (7, 8), while preterm birth, growth restriction, and gestational weight gain have also been linked to future cardiovascular disease (9-11). By comparison, relatively little is known about the long-term risk of maternal renal disease following complications of pregnancy.

There is some evidence to suggest that PE increases the risk of maternal kidney disease later in life. A meta-analysis of seven small prospective studies identified an increased risk of microalbuminuria among women who experienced PE compared with those who had normal pregnancies after a mean of seven years follow-up (12). A large Norwegian cohort study suggested that preeclamptic women were at long-term risk of ESKD (13). However, the proposed association may be complicated by the strong links between PE and unmeasured cardiovascular risk factors, such as maternal obesity, which can independently increase the risk of CKD (14).

The long-term risk of CKD among these women has not been comprehensively addressed by studies to date. It is biologically plausible that PE predisposes women to higher risk of CKD; PE is associated with abnormal placental development, and subsequent development of generalised maternal endothelial dysfunction (15). PE may thus result in lasting renal endothelial damage, which may increase maternal risk of CKD and ESKD (16). PE is just one of several hypertensive disorders of pregnancy (HDP), and it is unknown if women who experience gestational hypertension, or PE superimposed on chronic hypertension are also at increased long-term risk of maternal kidney disease.

A range of other complications of pregnancy may increase the risk of future kidney disease in women. Low birth weight and preterm delivery have been reported to increase the risk of ESKD in women with PE (13). However, it is uncertain whether these associations persist independently, or whether birth weight and gestational age are mediators of the association with PE. In one Norwegian cohort study, preterm birth was reported to independently increase the risk of ESKD, but this study was restricted to women with pre-existing diabetes and is not generalisable to the wider population (17).

GDM is an established risk factor for type 2 diabetes (18), and may thereby increase the risk of diabetic nephropathy. However, GDM is also an independent risk factor for vascular endothelial dysfunction (19, 20), and is a plausible independent risk factor for maternal kidney disease. To date there has been limited research to examine whether GDM predicts CKD risk independent of subsequent type 2 diabetes.

Worldwide, 3-5% of pregnancies are complicated by PE (21); 5-18% of births are preterm (22); 11% have low birth weight (23); and 5-13% are complicated by GDM (24, 25). Given the high prevalence of CKD (1), any true associations between these exposures and CKD may have potentially important public health implications, particularly in resource-poor settings where adverse pregnancy outcomes are more prevalent. Women with complications of pregnancy, who may be at risk of CKD, may benefit from future risk reduction interventions, or enhanced community-based follow-up care to mitigate against possible progression to renal disease. Only a minority of individuals with CKD will ever progress to ESKD, requiring dialysis or renal transplant, but they experience premature mortality (26, 27), and place a considerable economic and resource burden on health systems (28).

For peer review only - http://bmjopen.bmj.com/site/about/guidelines.xhtml

The aim of this systematic review and meta-analysis is to summarise the available evidence examining the association between adverse pregnancy outcomes and long-term maternal renal disease. The adverse outcomes of interest include HDP (including PE, gestational hypertension, chronic hypertension, and PE superimposed on chronic hypertension), preterm birth, delivery of a low birth weight infant, and GDM. The protocol has been registered on PROSPERO (https://www.crd.york.ac.uk/prospero/), registration number CRD42018110891.

Population

Women who have had at least one pregnancy of at least 23 weeks gestation

Exposures

Any one of the following adverse pregnancy outcomes:

1. Diagnosis of HDP (including PE, gestational hypertension, or other HDP)
2. Pre-term birth
3. Diagnosis of GDM
4. Delivery of a low birth weight infant (including infants who were small for gestational age)

For any of these adverse outcomes, they can be defined either using established clinical criteria, hospital records, or self-reporting of a doctor diagnosis.

Comparison

Women who never had a corresponding adverse outcome in pregnancy. For example, women who experienced a preterm birth in at least one pregnancy will be compared with women who never experienced a preterm birth.

Outcomes

Primary outcome 1: CKD

Primary outcome 2: ESKD

These primary outcomes can be defined either using established clinical criteria, or hospital records.

Secondary outcomes: (1) Hospitalisation for renal disease, (2) Deaths from renal disease

For peer review only

Review question

Does the presence of an adverse pregnancy outcome (i.e. HDP, preterm birth, delivery of a low birth weight infant, or GDM) increase the risk of maternal kidney disease in later life?

METHODS AND DESIGN

This protocol was drafted using the Preferred Reporting Items for Systematic Reviews and Meta-Analysis Protocols (PRISMA-P) checklist (29). The proposed systematic review and meta-analysis will follow the Meta-analyses Of Observational Studies in Epidemiology (MOOSE) guidelines (30).

Search strategy

The lead author (PB) will undertake a systematic search using the following databases: PubMed, EMBASE and Web of Science. Peer-reviewed journal articles published in English language, from inception of databases to 31 July 2018, will be included. A detailed search strategy has been compiled, and the search terms will be combined using Boolean Logic where appropriate (AND, OR). The detailed search strategy is available in Appendix 1.

Searches of the electronic databases will be supplemented by hand-searching all included papers to identify any further potentially relevant studies.

Study selection

The titles and abstracts of studies retrieved from each database search will be stored in EndNote reference manager, and de-duplicated. Two review authors (PB, SC) will screen all titles and abstracts for potentially relevant studies. The full text of relevant studies will then be retrieved and will be screened for compliance with eligibility criteria by two reviewers (PB, CJ). If consensus on eligibility cannot be achieved, a third review author will be consulted (AK). For any articles which do not meet inclusion criteria, the reasons for rejection will be noted. A MOOSE flow diagram documenting the process of study selection will be completed.

Inclusion criteria

- Case-control studies and cohort studies (prospective or retrospective).
- Data provided on an adverse pregnancy outcome of interest (i.e. diagnosis of HDP, preterm birth, low birth weight, or GDM) as an exposure variable.
- Data provided on a diagnosis of either CKD or ESKD as an outcome variable.
- Provides a measure of association between one or more of the adverse pregnancy outcomes and CKD or ESKD.

For peer review only - http://bmjopen.bmj.com/site/about/guidelines.xhtml

- The diagnosis of maternal CKD or ESKD is made at least three months after pregnancy has ended.
- Data must be from an original study
- Only English-language studies will be considered, including all years from inception of the electronic databases until July 2018.
- Peer-reviewed literature only will be included.

Exclusion criteria

- Non-human studies.
- Studies that are not in English.
- Studies focused on CKD/ESKD risk in the offspring.
- Studies focused on risk of maternal acute kidney injury, without reference to subsequent CKD/ESKD.
- Case reports, case series, letters, commentaries, notes, and editorials.
- Studies focused on women with pre-existing renal disease.

For peer review only

Data extraction

Data from all eligible studies will be extracted by two reviewers (PB, SC) using a standardised data collection form, including the author and year of publication, the study design, the exposure(s) and outcome(s) of interest, the definition used for each exposure and outcome, the stage/severity of the outcome (i.e. CKD), length of follow-up, the sample size, the confounders adjusted for (if any) and the crude and adjusted measures of association. Where necessary, corresponding authors of published studies will be contacted to obtain any information needed relating to effect estimates. Where effect estimates are not available, absolute numbers of events will be extracted, and crude measures of association will be calculated. If discrepancies arise in data extraction, these will be discussed between reviewers, and where necessary, a third reviewer will be consulted to achieve consensus (AK).

Quality appraisal of included studies

The quality of all included studies will be independently assessed by two reviewers using an established quality assessment tool for observational studies (PB, CJ). This tool has been described in detail elsewhere (31). Six types of bias will be assessed: selection, exposure, outcome, analytical, attrition, and confounding. For each study, each component will be assigned a risk of bias category: minimal, low, moderate, high, or not reported. For example, selection bias will be categorised as 'minimal' if the sample was from a 'consecutive unselected population', whereas selection bias will

be categorised as ‘high’ if sample selection is unclear and if the sample is not representative of the population of interest. For each included study, the overall likelihood of bias will be assessed and reported. Where discrepancies in quality appraisal arise, a third reviewer will be consulted to achieve consensus (AK).

Data synthesis and assessment for heterogeneity

Separate meta-analyses will be undertaken for each of the exposure variables and two primary outcomes where possible. Each meta-analysis will be undertaken to calculate the pooled estimate of the relationship between the adverse outcome of interest and subsequent development of maternal CKD or ESKD. For example, for preterm birth as the adverse outcome of interest, a meta-analysis will be undertaken to investigate the association between (1) preterm birth and CKD and (2) preterm birth and ESKD. For HDP, a meta-analysis will be undertaken to investigate the association between (1) any HDP and CKD and (2) any HDP and ESKD. Where possible, subgroup analyses will investigate the associations between PE, gestational hypertension, and any other HDP with each of the primary outcomes respectively. For delivery of infants with low birth weight, a meta-analysis will be undertaken to investigate the association between (1) low birth weight infant and maternal CKD and (2) low birth weight infant and maternal ESKD. Where possible, subgroup analyses will investigate the associations between small for gestational age infants with each of the primary outcomes respectively.

Both crude and adjusted effect estimates will be displayed using the generic inverse variance method. Adjustment will be based on the definition outlined in each of the individual studies. Heterogeneity will be explored based on I^2 values and chi-squared statistics. Random-effects models will be used if moderate or high levels of heterogeneity are observed between the studies of interest. If studies cannot be meaningfully combined in a meta-analysis, they will be presented in tabular format.

We will perform the following subgroup/sensitivity analyses where the data allow, using RevMan 5.3: (1) study type (case-control vs. cohort study), (2) stage/severity of CKD, (3) ethnic group, (4) length of follow-up after index pregnancy, (5) number of pregnancies affected by the adverse outcome, (6) measurement of exposure and outcome data (self-reported vs medical records vs laboratory measurements), (7) study quality (minimal/low risk of bias vs. moderate/high risk of bias. Furthermore, any studies which have not excluded women with pre-existing renal disease, or have not adjusted for this factor, will be considered in a separate subgroup analysis.

Where 10 or more studies are included in a meta-analysis, we will assess for publication bias. The trim and fill method will be used to identify and correct for funnel plot asymmetry arising from publication bias if appropriate (32).

Ethics and dissemination

This protocol is based on published data, and thus there is no requirement for ethics approval. The results will be disseminated through publication in a peer-reviewed journal, and through presentations at academic conferences.

For peer review only - http://bmjopen.bmj.com/site/about/guidelines.xhtml

Patient and public involvement

Patients were not involved in the design of this systematic review and meta-analysis. However, the authors will communicate the study findings to patient and public groups with an interest in this area, including Action on Pre-eclampsia.

Potential limitations

There are a number of limitations anticipated in this review. Publication bias may reduce the likelihood of retrieving studies which report non-significant associations between adverse pregnancy outcomes and maternal renal disease. Due to limited resources, only studies which are published in the English language will be included. Publications which only use biomarkers of renal function as outcome variables (e.g. microalbuminuria, albumin/creatinine ratio, estimated glomerular filtration rate) will not be included unless they are directly related to a diagnosis of CKD or ESKD.

A degree of heterogeneity is anticipated between studies. Differences in diagnostic methods are likely for both exposures and outcomes. The timeframe for follow-up is likely to vary considerably between studies, with those women followed up for longer more likely to have developed renal disease. The ability to identify any true associations between adverse pregnancy outcomes and future CKD or ESKD will be limited by the length of follow up in each of the included studies. Differences in sampling frames are also likely to lead to heterogeneity. Thus, a random effects model will be used for meta-analyses with moderate or high heterogeneity.

The presence of selection bias and residual confounding is a concern in all observational studies. Potential confounders may include maternal age, ethnic group, socio-economic status, parity, family history, hypertension, diabetes, cardiovascular disease, systemic inflammatory disease, hyperlipidaemia, obesity, smoking. Our meta-analyses will display both crude and adjusted results where possible, basing adjustment on the definition outlined in each individual study. However, given that less adjusted effect estimates may skew the overall results, a sensitivity analysis will be undertaken where possible to examine effect estimates which are adjusted more fully for confounders (i.e. adjusted for, at a minimum, maternal age, hypertension, diabetes, obesity, smoking, pre-existing kidney disease).

For peer review only

DISCUSSION

There is a lack of consensus on whether adverse pregnancy outcomes, such as HDP, preterm birth, delivery of a low birth-weight infant, and GDM, independently increase the risk of maternal CKD and ESKD. This systematic review and meta-analysis will summarise the available evidence which has examined these associations, thus providing novel information on the role of pregnancy-related factors in the aetiology of maternal renal disease.

ACKNOWLEDGEMENTS

For peer review only - http://bmjopen.bmj.com/site/about/guidelines.xhtml

Competing interests

None declared

Funding

This work was performed within the Irish Clinical Academic Training (ICAT) Programme, supported by the Wellcome Trust and the Health Research Board (Grant Number 203930/B/16/Z), the Health Service Executive National Doctors Training and Planning and the Health and Social Care, Research and Development Division, Northern Ireland.

Author contributions

PB, FMcC, IP and ASK conceived and designed the protocol, and PB drafted the protocol manuscript. PB developed the search strategy, with input from FMcC, IP and ASK. PB, CJ and ASK planned the data extraction. PB, SC and ASK planned the quality appraisal of all included studies. PB, FMcC, IP, SC, CJ, KK, MK, ME, PS and ASK critically revised the manuscript for methodological and intellectual content. All authors approved the final version.

For peer review only

REFERENCES

1. Hill NR, Fatoba ST, Oke JL, Hirst JA, O'Callaghan CA, Lasserson DS, et al. Global Prevalence of Chronic Kidney Disease - A Systematic Review and Meta-Analysis. PloS one. 2016;11(7):e0158765.
2. Couser WG, Remuzzi G, Mendis S, Tonelli M. The contribution of chronic kidney disease to the global burden of major noncommunicable diseases. Kidney Int. 2011;80(12):1258-70.
3. Eggers PW. Has the incidence of end-stage renal disease in the USA and other countries stabilized? Curr Opin Nephrol Hypertens. 2011;20(3):241-5.
4. Gillon TE, Pels A, von Dadelszen P, MacDonell K, Magee LA. Hypertensive disorders of pregnancy: a systematic review of international clinical practice guidelines. PloS one. 2014;9(12):e113715.
5. Jeyabalan A. Epidemiology of preeclampsia: impact of obesity. Nutr Rev. 2013;71 Suppl 1:S18-25.
6. Di Cianni G, Miccoli R, Volpe L, Lencioni C, Del Prato S. Intermediate metabolism in normal pregnancy and in gestational diabetes. Diabetes/metabolism research and reviews. 2003;19(4):259-70.
7. Mosca L, Benjamin EJ, Berra K, Bezanson JL, Dolor RJ, Lloyd-Jones DM, et al. Effectiveness-based guidelines for the prevention of cardiovascular disease in women--2011 update: a guideline from the American Heart Association. Journal of the American College of Cardiology. 2011;57(12):1404-23.
8. Bushnell C, McCullough L. Stroke prevention in women: synopsis of the 2014 American Heart Association/American Stroke Association guideline. Annals of internal medicine. 2014;160(12):853-7.
9. van Kesteren F, Visser S, Hermes W, Teunissen PW, Franx A, van Pampus MG, et al. Counselling and management of cardiovascular risk factors after preeclampsia. Hypertension in pregnancy. 2016;35(1):55-61.
10. Cirillo PM, Cohn BA. Pregnancy complications and cardiovascular disease death: 50-year follow-up of the Child Health and Development Studies pregnancy cohort. Circulation. 2015;132(13):1234-42.
11. Heida KY, Velthuis BK, Oudijk MA, Reitsma JB, Bots ML, Franx A, et al. Cardiovascular disease risk in women with a history of spontaneous preterm delivery: A systematic review and meta-analysis. Eur J Prev Cardiol. 2016;23(3):253-63.
12. McDonald S, Han Z, Michael W, Gerstein H, Devereaux P. Renal disease after preeclampsia: A systematic review and meta-analyses. Pregnancy Hypertension. 2010;1:S41.
13. Vikse BE, Irgens LM, Leivestad T, Skjaerven R, Iversen BM. Preeclampsia and the risk of end-stage renal disease. The New England journal of medicine. 2008;359(8):800-9.
14. Lastra G, Manrique C, Sowers JR. Obesity, cardiometabolic syndrome, and chronic kidney disease: the weight of the evidence. Adv Chronic Kidney Dis. 2006;13(4):365-73.
15. Zhou Y, Damsky CH, Fisher SJ. Preeclampsia is associated with failure of human cytotrophoblasts to mimic a vascular adhesion phenotype. One cause of defective endovascular invasion in this syndrome? The Journal of clinical investigation. 1997;99(9):2152-64.
16. Rangaswami J, Naranjo M, McCullough PA. Preeclampsia as a Form of Type 5 Cardiorenal Syndrome: An Underrecognized Entity in Women's Cardiovascular Health. Cardiorenal Med. 2018;8(2):160-72.
17. Sandvik MK, Iversen BM, Irgens LM, Skjaerven R, Leivestad T, Softeland E, et al. Are adverse pregnancy outcomes risk factors for development of end-stage renal disease in women with diabetes? Nephrology, dialysis, transplantation : official publication of the European Dialysis and Transplant Association - European Renal Association. 2010;25(11):3600-7.
18. Bellamy L, Casas JP, Hingorani AD, Williams D. Type 2 diabetes mellitus after gestational diabetes: a systematic review and meta-analysis. Lancet (London, England). 2009;373(9677):1773-9.

For peer review only - http://bmjopen.bmj.com/site/about/guidelines.xhtml

19. Knock GA, McCarthy AL, Lowy C, Poston L. Association of gestational diabetes with abnormal maternal vascular endothelial function. British journal of obstetrics and gynaecology. 1997;104(2):229-34.
20. Lappas M. Markers of endothelial cell dysfunction are increased in human omental adipose tissue from women with pre-existing maternal obesity and gestational diabetes. Metabolism: clinical and experimental. 2014;63(6):860-73.
21. Hypertension in pregnancy. Report of the American College of Obstetricians and Gynecologists' Task Force on Hypertension in Pregnancy. Obstetrics and gynecology. 2013;122(5):1122-31.
22. Blencowe H, Cousens S, Oestergaard MZ, Chou D, Moller AB, Narwal R, et al. National, regional, and worldwide estimates of preterm birth rates in the year 2010 with time trends since 1990 for selected countries: a systematic analysis and implications. Lancet (London, England). 2012;379(9832):2162-72.
23. The World Bank. Low-birthweight babies (% of births) 2018 [Available from: https://data.worldbank.org/indicator/SH.STA.BRTW.ZS.
24. Eades CE, Cameron DM, Evans JMM. Prevalence of gestational diabetes mellitus in Europe: A meta-analysis. Diabetes research and clinical practice. 2017;129:173-81.
25. Zhu Y, Zhang C. Prevalence of Gestational Diabetes and Risk of Progression to Type 2 Diabetes: a Global Perspective. Current diabetes reports. 2016;16(1):7.
26. Tonelli M, Wiebe N, Culleton B, House A, Rabbat C, Fok M, et al. Chronic kidney disease and mortality risk: a systematic review. J Am Soc Nephrol. 2006;17(7):2034-47.
27. Di Angelantonio E, Chowdhury R, Sarwar N, Aspelund T, Danesh J, Gudnason V. Chronic kidney disease and risk of major cardiovascular disease and non-vascular mortality: prospective population based cohort study. BMJ. 2010;341:c4986.
28. Levin A, Tonelli M, Bonventre J, Coresh J, Donner JA, Fogo AB, et al. Global kidney health 2017 and beyond: a roadmap for closing gaps in care, research, and policy. Lancet (London, England). 2017;390(10105):1888-917.
29. Moher D, Shamseer L, Clarke M, Ghersi D, Liberati A, Petticrew M, et al. Preferred reporting items for systematic review and meta-analysis protocols (PRISMA-P) 2015 statement. Systematic reviews. 2015;4:1.
30. Stroup DF, Berlin JA, Morton SC, Olkin I, Williamson GD, Rennie D, et al. Meta-analysis of observational studies in epidemiology: a proposal for reporting. Meta-analysis Of Observational Studies in Epidemiology (MOOSE) group. Jama. 2000;283(15):2008-12.
31. McDonald SD, Han Z, Mulla S, Murphy KE, Beyene J, Ohlsson A. Preterm birth and low birth weight among in vitro fertilization singletons: a systematic review and meta-analyses. European journal of obstetrics, gynecology, and reproductive biology. 2009;146(2):138-48.
32. Weinhandl ED, Duval S. Generalization of trim and fill for application in meta-regression. Research synthesis methods. 2012;3(1):51-67.

For peer review only

For peer review only - http://bmjopen.bmj.com/site/about/guidelines.xhtml

For peer review only

For peer review only - http://bmjopen.bmj.com/site/about/guidelines.xhtml

SUPPLEMENTARY FILE – Search Strategy

Preeclampsia & gestational hypertension

H1	Antenatal
H2	Ante-natal
H3	Prenatal
H4	Pre-natal
H5	Pregnan*
H6	Gestation*
H7	H1 OR H2 OR H3 OR H4 OR H5 OR H6
H8	Pre Eclampsia
H9	Preeclampsia
H10	Pre-eclampsia
H11	Eclampsia
H12	Edema-Proteinuria-Hypertension
H13	Edema Proteinuria Hypertension
H14	Oedema-Proteinuria-Hypertension
H15	Oedema Proteinuria Hypertension
H16	EPH
H17	Toxemia*
H18	Toxaemia*
H19	Gestosis
H20	(H19 OR H7)
H21	Toxemia* OR Toxaemia* or EPH
H22	H21 AND H20
H23	Gestational hypertension
H24	Pregnancy-induced hypertension
H25	Pregnancy induced hypertension
H26	Hypertensi*
H27	Blood pressure
H28	H26 OR H27
H29	H28 AND H7
H30	H8 OR H9 OR H10 OR H11 OR H12 OR H13 OR H14 OR H15 OR H22 OR H23 OR H24 OR H25 OR H29

Gestational diabetes

D1	Pregnancy-Induced diabetes
D2	Pregnancy Induced diabetes
D3	Gestational Diabetes
D4	diabetes in pregnancy
D5	diabetes of pregnancy
D6	Diabetes mellitus in pregnancy
D7	Diabetes mellitus of pregnancy
D8	pregnancy-related diabetes
D9	pregnancy related diabetes
D10	D1 OR D2 OR D3 OR D4 OR D5 OR D6 OR D7 OR D8 OR D9 OR D10

For peer review only - http://bmjopen.bmj.com/site/about/guidelines.xhtml

Low birth weight

W1	Low Birth Weight
W2	Low-Birth-Weight
W3	Low Birth Weights
W4	Low-Birth-Weights
W5	Low Birthweight
W6	Low Birthweights
W7	Extremely Low Birth Weight
W8	Extremely-Low-Birth-Weight
W9	Extremely-Low-Birth-Weights
W10	Extremely Low Birthweight
W11	Very Low Birth Weight
W12	Very-Low-Birth-Weight
W13	Very-Low-Birth-Weights
W14	Very Low Birthweight
W15	Birth Weights
W16	Birth Weight
W17	Birthweight
W18	LBW
W19	VLBW
W20	ELBW
W21	Small for gestational age
W22	Growth restrict*
W23	IUGR
W24	W1 OR W2 OR W3 OR W4 OR W5 OR W6 OR W6 OR W7 OR W8 OR W9 OR W10 OR W11 OR W12 OR W13 OR W14 OR W15 OR W16 OR W17 OR W18 OR W19 OR W20 OR W21 OR W22 OR W23

Preterm birth or SGA

P1	Premature Birth
P2	Preterm Birth
P3	Premature Births
P4	Preterm Births
P5	Pre-term Birth
P6	Pre-term Births
P7	Gestational age
P8	Gestational ages
P9	Fetal Age
P10	Foetal Age
P11	Fetal Ages
P12	Foetal Ages
P13	Premature Labor
P14	Preterm Labor
P15	Pre-term Labor
P16	Premature Labour
P17	Preterm Labour
P18	Pre-term Labour
P19	Premature Obstetric Labor
P20	Premature Obstetric Labour
P21	P1 OR P2 OR P3 OR P4 OR P5 OR P6 OR P7 OR P8 OR P9 OR P10 OR P11 OR P12 OR P13 OR P14 OR P15 OR P16 OR P17 OR P18 OR P19 OR P20

For peer review only - http://bmjopen.bmj.com/site/about/guidelines.xhtml

Women

WO1	Woman
WO2	Women
WO3	Female*
WO4	Mother*
WO5	maternal
WO6	WO1 OR WO2 OR WO3 OR WO4 OR WO5

Chronic or End-stage kidney disease

K1	Chronic
K2	Long-term
K3	Long term
K4	End-stage
K5	End stage
K6	K1 OR K2 OR K3 OR K4 OR K5
K7	Kidney*
K8	renal
K9	K7 OR K8
K10	Insufficien*
K11	Disease*
K12	Fail*
K13	Impair*
K14	Dysfunction*
K15	K10 OR K11 OR K12 OR K13 OR K14
K16	K6 AND K9 AND K15
K17	CKD
K18	CRD
K19	CKF
K20	CRF
K21	ESKD
K22	ESRD
K23	ESKF
K24	ESRF
K25	K17 OR K18 OR K19 OR K20 OR K21 OR K22 OR K23 OR K24
K26	K16 OR K25

Search strategy

(H30 OR D10 OR W24 OR P21) AND WO6 AND K26

For peer review only - http://bmjopen.bmj.com/site/about/guidelines.xhtml

For peer review only

PRISMA-P (Preferred Reporting Items for Systematic review and Meta-Analysis Protocols) 2015 checklist: recommended items to address in a systematic review protocol*

Section and topic	Item No	Checklist item	(Page No.#)
**ADMINISTRATIVE INFORMATION**			
Title:			
Identification	1a	Identify the report as a protocol of a systematic review	1
Update	1b	If the protocol is for an update of a previous systematic review, identify as such	n/a
Registration	2	If registered, provide the name of the registry (such as PROSPERO) and registration number	5
Authors:			
Contact	3a	Provide name, institutional affiliation, e-mail address of all protocol authors; provide physical mailing address of corresponding author	1
Contributions	3b	Describe contributions of protocol authors and identify the guarantor of the review	10
Amendments	4	If the protocol represents an amendment of a previously completed or published protocol, identify as such and list changes; otherwise, state plan for documenting important protocol amendments	n/a
Support:			
Sources	5a	Indicate sources of financial or other support for the review	10
Sponsor	5b	Provide name for the review funder and/or sponsor	10
Role of sponsor or funder	5c	Describe roles of funder(s), sponsor(s), and/or institution(s), if any, in developing the protocol	10
**INTRODUCTION**			
Rationale	6	Describe the rationale for the review in the context of what is already known	4
Objectives	7	Provide an explicit statement of the question(s) the review will address with reference to participants, interventions, comparators, and outcomes (PICO)	4, 5
**METHODS**			
Eligibility criteria	8	Specify the study characteristics (such as PICO, study design, setting, time frame) and report characteristics (such as years considered, language, publication status) to be used as criteria for eligibility for the review	5, 6, 7
Information sources	9	Describe all intended information sources (such as electronic databases, contact with study authors, trial registers or other grey literature sources) with planned dates of coverage	6, 7
Search strategy	10	Present draft of search strategy to be used for at least one electronic database, including planned limits, such that it could be repeated	13-15

For peer review only

Study records:			
Data management	11a	Describe the mechanism(s) that will be used to manage records and data throughout the review	6
Selection process	11b	State the process that will be used for selecting studies (such as two independent reviewers) through each phase of the review (that is, screening, eligibility and inclusion in meta-analysis)	6
Data collection process	11c	Describe planned method of extracting data from reports (such as piloting forms, done independently, in duplicate), any processes for obtaining and confirming data from investigators	7
Data items	12	List and define all variables for which data will be sought (such as PICO items, funding sources), any pre-planned data assumptions and simplifications	7
Outcomes and prioritization	13	List and define all outcomes for which data will be sought, including prioritization of main and additional outcomes, with rationale	5
Risk of bias in individual studies	14	Describe anticipated methods for assessing risk of bias of individual studies, including whether this will be done at the outcome or study level, or both; state how this information will be used in data synthesis	7, 8
Data synthesis	15a	Describe criteria under which study data will be quantitatively synthesised	8
	15b	If data are appropriate for quantitative synthesis, describe planned summary measures, methods of handling data and methods of combining data from studies, including any planned exploration of consistency (such as I^2, Kendall's τ)	8
	15c	Describe any proposed additional analyses (such as sensitivity or subgroup analyses, meta-regression)	8
	15d	If quantitative synthesis is not appropriate, describe the type of summary planned	8
Meta-bias(es)	16	Specify any planned assessment of meta-bias(es) (such as publication bias across studies, selective reporting within studies)	8
Confidence in cumulative evidence	17	Describe how the strength of the body of evidence will be assessed (such as GRADE)<br><br>***Only observational studies will be included. Strength of evidence will be based on quality assessment and adjustment for confounders. If possible, a hierarchy of adjustment will be used in sensitivity analysis*	7, 8**

*** It is strongly recommended that this checklist be read in conjunction with the PRISMA-P Explanation and Elaboration (cite when available) for important clarification on the items. Amendments to a review protocol should be tracked and dated. The copyright for PRISMA-P (including checklist) is held by the PRISMA-P Group and is distributed under a Creative Commons Attribution Licence 4.0.**

From: Shamseer L, Moher D, Clarke M, Ghersi D, Liberati A, Petticrew M, Shekelle P, Stewart L, PRISMA-P Group. Preferred reporting items for systematic review and meta-analysis protocols (PRISMA-P) 2015: elaboration and explanation. BMJ. 2015 Jan 2;349(jan02 1):g7647.

For peer review only
